# Knowledge and future preference of Chinese women in a major public hospital in Hong Kong after undergoing non-invasive prenatal testing for positive aneuploidy screening: a questionnaire survey

**DOI:** 10.1186/s12884-015-0636-7

**Published:** 2015-09-02

**Authors:** Kam On Kou, Chung Fan Poon, Wai Ching Tse, Shui Lam Mak, Kwok Yin Leung

**Affiliations:** Department of Obstetrics and Gynaecology, Queen Elizabeth Hospital, 30 Gascoigne Road, Hong Kong, SAR China

## Abstract

**Background:**

Despite the non-invasive nature of non-invasive prenatal testing (NIPT), there is still a need for a separate informed consent process before testing. The objectives of this study are to assess (a) knowledge and preferences of Chinese women in a major public hospital in Hong Kong who underwent NIPT, and (b) whether their knowledge and preferences differ depending on womens’ characteristics and sources of information.

**Methods:**

Setting: prenatal diagnosis and counselling clinic.

Between February 2012 and September 2013, a questionnaire survey was distributed to all women who underwent NIPT after positive aneuploidy screening. As a pilot study, ten knowledge questions were designed based on the rapid response statement on Prenatal Detection of Down Syndrome using Massively Parallel Sequencing from the International Society for Prenatal Diagnosis in 2011. The source of women’s knowledge and their preferences were also evaluated. While conventional screening was publicly funded, NIPT was not. Differences between subgroups were compared using chi square tests and logistic regression analysis.

**Results:**

Of 152 women who underwent NIPT, 135 (88.8 %) completed their questionnaires. More than 90 % of women recognised the possibility of false positive and false negative results. Slightly more than 70 % of women knew the inferior sensitivity of NIPT compared to an invasive test, and the possibility of an uninformative test result, but were not aware of the complicated aspects of NIPT. Pregnant women with an advanced level of education or those who underwent NIPT before 15 weeks provided answers that was more accurate by around 10-20 % in two to three knowledge questions than those without. These associations were confirmed by multivariate logistic regression analysis. The women received information on NIPT largely from their private doctors (47.4 %) and web (41.5 %). In their future pregnancies, more women would opt for NIPT (a self-financed item) after positive screening (‘free’ in a public hospital) (57.8 %) than as a primary screening (30.4 %).

**Conclusions:**

It is feasible to use a questionnaire based on the ISPD statement on NIPT to assess women’s knowledge of the test. The Chinese women who underwent NIPT recognised the limitations, but did not understand the complicated aspects. More information should be provided by health care professionals in order to facilitate an informed choice by patients. More women preferred NIPT as a contingent test than as a primary screening probably because of its high cost.

## Background

Among various prenatal screening methods for Down syndrome, non-invasive prenatal testing (NIPT), a new technology assessing cell-free fetal DNA (cffDNA) in maternal blood, is the most sensitive and specific one [1–8]. Its recent introduction resulted in a marked decline in the number of invasive procedures [9–13], and conventional screening [10–12], even in the first year of its availability. NIPT is well accepted by women [10, 11, 13, 14] and offered by obstetricians [15].

Despite the non-invasive nature of NIPT, both pregnant women [16] and genetic counsellors [17] regard that there is still a need for a separate informed consent process before testing. Major governing or professional bodies including the American College of Obstetricians and Gynecologists, the American College of Medical Genetics, National Society of Genetic Counselors (NSGC), and International Society for Prenatal Diagnosis (ISPD) recommend offering NIPT in the context of informed consent, education and counselling [18–21]. Whether women are given adequate information before making an informed decision for NIPT is not known. Besides, whether a previous history of NIPT affects women’s preference for NIPT in the future pregnancy is also not known. NIPT was introduced in Hong Kong in August 2011. Few studies have been conducted on women’s knowledge and preferences. The objectives of this study are to assess (a) knowledge and preferences of Chinese women in a major public hospital in Hong Kong who underwent NIPT, and (b) whether their knowledge and preferences differ depending on their characteristics and sources of information.

## Methods

Between February 2012 and September 2013, we invited all Chinese pregnant women who underwent NIPT for a positive conventional aneuploidy screening to complete a questionnaire in the setting of our prenatal diagnosis and counselling clinic. All included women underwent Down syndrome screening at our clinic. Screen-positive women who underwent NIPT were followed up for counselling and a mid-trimester anomaly scan during which the questionnaire was completed. Our hospital is one of the largest public hospitals in Hong Kong, with more than 6,000 deliveries a year. Multiple pregnancies were excluded. The study protocol was approved by the Research Ethics Committee (Kowloon Central/Kowloon East), Hospital Authority, Hong Kong. This research project conforms to the provisions of the Declaration of Helsinki. Informed consent was obtained from every participant in this study.

### Options for prenatal testing for Down syndrome

Since 1 July 2010, universal prenatal screening for Down syndrome has been offered to all pregnant women after adequate counselling irrespective of maternal age. The options included first trimester combined (based on nuchal translucency, pregnancy-associated plasma protein A (PAPP-A) and free beta human chorionic gonadotropin (beta hCG)), or second trimester maternal serum screening (based on alpha fetoprotein and total beta hCG) depending on the week of gestation at the time of booking. All counselling on screen-positive women were conducted or supervised by a maternal and fetal medicine subspecialist, and different options including an invasive test or no further testing were discussed. Since August 2011, NIPT has been available in the private sector in Hong Kong, and this option was also discussed as an alternative to an invasive test with screen-positive women in our clinic upon questioning. Most commercial NIPT tests during the study period were based on massively parallel sequencing with ‘shortgun’ counting of all cell-free DNA sequences, while the remaining used ‘targeted’ counting of specific DNA sequences. An information sheet on NIPT including the process, benefits and limitations was provided. After undergoing NIPT with separate counselling provided by private obstetricians, women were followed up, rescanned for fetal structures, counselling on the NIPT result, and offered a free option of invasive testing after a positive NIPT result or for other reasons. As a pilot study, a questionnaire was designed to assess the quality of counselling that we provided.

### Questionnaire on knowledge and preference

The questionnaire was designed to assess women’s characteristics, knowledge on NIPT and preference in future pregnancies. Demographic factors including age, gravidity, parity, method of conception, education, employment, and gestational week whilst undergoing NIPT were collected. There were ten knowledge questions based on the rapid response statement on Prenatal Detection of Down Syndrome using Massively Parallel Sequencing from a committee on behalf of the Board of the International Society for Prenatal Diagnosis (ISPD) in October 2011^17^ (Table 2). The source (doctor, web, books, colleagues, friends or others) of their knowledge on NIPT was also assessed. The questionnaire was printed, distributed to the women in our prenatal diagnosis and counselling clinic and completed by them with the assistance of an experienced midwife if required, to explain medical terms or general terms in a straightforward manner if Cantonese was not their mother tongue.

### Statistical analysis

With the use of descriptive statistics, the proportions of respondents selecting each answer to questions on the knowledge and preference were presented. Their answers on knowledge questions were then recorded as correct or incorrect. We analysed whether their knowledge and preference on NIPT vary with demographic factors and source of information using chi square test and logistic regression with backward stepwise (likelihood ratio) analysis. All statistical analysis was performed using SPSS 16.0 for Windows.

## Results

Between August 2011 and July 2013, 152 women underwent NIPT. The results and impact on the number of invasive testing were presented previously [13]. Two of them underwent invasive testing because of high-risk results for trisomy 21 or trisomy 18, and all positive results were confirmed. One woman underwent amniocentesis despite low-risk result for aneuploidy, and had a normal karyotype. All babies screened to be low risk by NIPT were confirmed normal after birth on physical examination.

Of these 152 women, 135 (88.8 %) completed the questionnaire. There were no differences in the characteristics between the respondents and non-respondents (Table 1). All 135 women filled out the questionnaire after learning the results of their NIPT. The mean interval between NIPT and questionnaire was 30.0 days. Of these 135 women who completed the questionnaire, 63.7 % were aged 35 or above, 91.1 % conceived naturally, 60.7 % were nulliparous, 66.7 % had a tertiary education or higher, and 86.7 % were employed (Table 1).

**Table 1:** Socio-demographic and obstetric characteristics of the women who underwent non-invasive prenatal testing (NIPT). n (%)

Characteristic	Respondents (n = 135)	Non-respondents (n = 16) ^a^	P value
Advanced maternal age (> = 35)	86 (63.7 %)	8 (50.0 %)	0.415
Parity			
0	82 (60.7)	9 (56.3 %)	0.685
1	49 (36.3 %)	7 (43.7 %)	
2	4 (3.0 %)	0 (0 %)	
Previous miscarriage or termination of pregnancy			
0	83 (61.5 %)	9 (56.3 %)	0.107
1	40 (29.6 %)	5 (31.3 %)	
2 or above	12 (8.9 %)	2 (12.5 %)	
Conceived naturally	123 (91.1 %)	16 (100 %)	0.366
Advanced education (tertiary education or above)	90 (66.7 %)	14 (87.5 %)	0.15
Employed	117 (86.7 %)	13 (81.3 %)	0.466
Underwent NIPT before 15 weeks	132 (97.8 %)	14 (87.5 %)	0.086

^a^one missing data

### Women’s knowledge on NIPT

More than 90 % of women recognised the potential for false positive and false negative results of NIPT for Down syndrome (Questions 2 and 3, Table 2). Around 95.6 % of women understood it would be very stressful if NIPT showed a positive result (Question 4, Table 2). More than 70 % of women recognised the inferior sensitivity of NIPT for the detection of chromosomal abnormalities compared to an invasive test, and additionally recognised the possibility of uninformative test results (Questions 1 and 5, Table 2).

**Table 2:** Responses of women to ten knowledge questions and two preferences questions on non-invasive prenatal testing (NIPT) (n = 135)

Questions	Yes	No	Do not know
1. NIPT can detect only half of the fetal chromosomal abnormalities that would be identified through amniocentesis or chorionic villus sampling	101 (74.8 %)^a^	13 (9.6 %)	21 (15.6 %)
2. NIPT does not detect all cases of fetal Down syndrome	124 (91.9 %)^a^	3 (2.2 %)	8 (5.9 %)
3. There are also occasional false-positive results and therefore women with positive NIPT results need to receive confirmatory testing through chorionic villus sampling or an amniocentesis	122 (90.4 %)^a^	5 (3.7 %)	8 (5.9 %)
4. Women with positive NIPT results are at very high risk of Down syndrome and for some women the extended period awaiting confirmatory invasive testing results is likely to be highly stressful	129 (95.6 %)^a^	1 (0.7 %)	5 (3.7 %)
5. For some women, a NIPT test result may not be informative because of inadequate amount of fetal DNA in maternal plasma or other reasons	97 (71.9 %)^a^	3 (2.2 %)	35 (25.9 %)
6. NIPT is suitable for those women who have or with a family history of a chromosomal abnormality carrying an increased risk of inheritance to their child	65 (48.2 %)	6 (4.4 %)^a^	64 (47.4 %)
7. NIPT is suitable for multiple pregnancies	73 (54.1 %)	7 (5.2 %)^a^	55 (40.7 %)
8. NIPT is suitable for pregnancies conceived after donor in vitro fertilization	56 (41.5 %)	3 (2.2 %)^a^	76 (56.3 %)
9. NIPT is suitable to detect fetal single gene disorder such as thalassemia	58 (43.0 %)	5 (3.7 %)^a^	72 (53.3 %)
10. NIPT is suitable for those women who recently received blood transfusion, organ transplant or stem cell therapy	55 (40.7 %)	7 (5.2 %)^a^	73 (54.1 %)
11. In your next pregnancy, you will opt for a conventional screening test for Down syndrome first and then, if screened high risk, will opt for a NIPT.	78 (57.8 %)	38 (28.1 %)	19 (14.1 %)
12. In your next pregnancy, you will opt for NIPT directly without undergoing a conventional screening test for Down syndrome.	41 (30.4 %)	73 (54.1 %)	21 (15.5 %)

Questions 1 to 10 were the ten knowledge questions developed on the basis of the rapid response statement of the Board of the International Society for Prenatal Diagnosis. Correct answers were marked as ^a^

For the knowledge questions on NIPT’s applicability to special at-risk groups (Questions 6–10, Table 2), about half of the women answered ‘do not know’ and less than 10 % answered correctly (marked ^a^ in Table 2). So, further analysis by stratification was not performed on answers to questions 6–10.

### Women’s knowledge and characteristics and information

The women received information on NIPT largely from their private doctors (47.4 %), web (41.5 %), relatives/friends (23.0 %), but little from their colleagues (3.0 %), books or magazines (0.7 %), or other means (5.2 %). Twenty-nine women (21.5 %) received information from more than one source.

Pregnant women who underwent NIPT before 15 weeks of gestation had significantly higher proportions of correct answers to three knowledge questions on NIPT with odds ratios of 6.2, 3.4 and 5.6 in Questions 2, 3 and 4 respectively than those after 15 weeks (Table 3). Besides, pregnant women with an advanced level of education had significantly higher proportions of correct answers in two knowledge questions on NIPT with odds ratios of 5.6 and 13.6 in Questions 2 and 4 respectively than those without (Table 3).

**Table 3:** Proportions and adjusted odds ratios (OR) of correct answers to the first five knowledge questions: comparing non-invasive prenatal testing (NIPT) before 15 weeks versus after 15 weeks and with advanced education versus without

	NIPT before 15 weeks				Advanced education			
Questions	Yes (%)	No (%)	*P* value	OR (95 % CI)	Yes (%)	No (%)	*P* value	OR (95 % CI)
1. Comparison with invasive test	71.5	62.5	0.125	2.1 (0.8–5.3)	77.8	68.9	0.262	1.5 (0.7–3.3)
2. Not 100 % detection	95.1	75.0	0.001*	6.2 (1.6–23.7)*	96.7	82.2	0.004*	5.6 (1.3–23.1)*
3. False-positive results	92.8	79.2	0.04*	3.4 (1.0–11.5)*	92.2	86.7	0.302	1.0 (0.6–6.3)
4. Stressful if positive	97.3	87.5	0.035*	5.6 (0.9–33.8)	98.9	88.9	0.008*	13.6 (1.5–125.5)*
5. May not be informative	74.8	58.5	0.104	2.1 (0.9-5.3)	75.6	64.4	0.176	1.6 (0.7–3.5)

*statistically significant

There were no significant differences in the proportions of correct answers to questions 1 to 5 according to their characteristics (including advanced maternal age, parity, history of miscarriage or termination of pregnancy, method of conception, status of employment) or source of information (including private doctors, web, relatives/friends, colleagues, books or magazines, and other means).

### Women’s preference

Significantly more women preferred NIPT as a contingency test than as a primary screening test in future pregnancies (57.8 % vs 30.4 %, p < 0.001). Subgroup analysis revealed no significant differences in preferences among women with different characteristics or different sources of information (as above).

## Discussion

In the present study, the majority (>90 %) of pregnant women recognised the possibilities of false negative and false positive results of NIPT, while more than 70 % of them recognised its limited sensitivity of detecting uncommon aneuploidies compared with an invasive test, and the possibility of uninformative test results. Only a small proportion (<10 %) of women answered correctly to questions on the applicability of NIPT in special groups of pregnant women. We did not identify any similar studies for comparisons. However, our findings are in accordance with previous studies that pregnant women generally know more about practical aspects of Down syndrome than screening limitations and accuracy [22, 23].

Pregnant women with an advanced level of education had higher proportions of correct answers in two knowledge questions on NIPT than those without. This finding is consistent with previous studies that education level predicted knowledge level on conventional screening for Down syndrome [24–26]. In addition, pregnant women who underwent NIPT before 15 weeks of gestation had higher proportions of correct answers in two knowledge questions on NIPT than those after 15 weeks. This result is not unexpected, as first-trimester screening is associated with the use of NIPT [13, 27]. We postulate that women with an earlier NIPT are more willing to know more about the test. Although Nullipara and employed women are associated with the use of NIPT, our present study did not show that these subgroups of women knew more about NIPT than Multipara and the unemployed. It seems that the need for adequate pre-test counselling of women is not limited to particular subgroups [28].

More women preferred conventional screening before NIPT to direct NIPT (57.8 % vs 30.4 %) even though they had experience with the forme. Women were most concerned with the cost of a screening test [29] besides the risk of miscarriage [30]. We postulate that the difference in their preferences was related to the price difference between a ‘free’ conventional screening offered by a public hospital and an expensive self-financed NIPT. However, if the price of NIPT decreases to an acceptable level in the future, women’s preference will probably change to opting for NIPT as a primary screening.

Consistent with a previous study [28], the women’s preference or self-risk assessment was independent of maternal age, education or parity. Less than half of the women replied that their information on NIPT was received from their private doctors, while nearly half of women learned it from the web. Self-risk assessment was influenced largely by counselling by health-care professionals and the media apart from personal experience [28].

The web is a common way of searching for information nowadays, but the accuracy of information on the web is variable. No specific healthcare profession most suited to providing counselling on NIPT has been identified. ACMG and NSGC recommend counselling by a qualified prenatal care provider, a trained designee or a genetic counsellor [20, 21]. In a study in U.K., women preferred pre- and post-NIPT counselling by a midwife [14]. In Hong Kong, trained midwives in maternal and fetal medicine team have been providing prenatal counselling on universal conventional Down syndrome screening to all pregnant women. This provides a good platform for further training on NIPT counselling. Continuous education is required to equip obstetricians and midwives with the competences in providing such counselling [31]. In a previous study on conventional screening tests, obstetricians would prefer waiting longer and accepting a greater decrease in detection rate for a safer test than midwives [32]. However, another study showed no correlation between individual healthcare professionals’ attitudes and screening uptake [33]. The determinants of the uptake rate of a diagnostic test are patient-centred after adequate counselling is provided [34].

NIPT should be offered in the context of informed decision making, education, pre-test and post-test, and counselling [18–21, 35]. In a previous study, around 72 % of women declined NIPT [35]. In the present study, around half the women preferred conventional screening despite a previous history of NIPT. High-risk women electing NIPT tend to have higher scores of depression/anxiety [36]. An increased level of knowledge of prenatal screening for Down syndrome was associated with less ambivalence and higher level of wellbeing, but not with worries about the results [37]. The information given should include the purpose, advantages, follow-up invasive testing, and limitations of NIPT [20]. Despite a risk of about 5 % of an uninformative test result of NIPT [35], about 30 % of the women in the present study were not aware of this possibility. Uninformative test results could lead to a delay in diagnosis or unavailability of information for risk assessment [20]. Besides, about 30 % of women were not aware of the limitation of NIPT in the detection of uncommon aneuploidies compared to an invasive test. It is important to correct false assumptions that might otherwise lead to these tests being declined by patients [25].

A major strength of this study was that the ten knowledge questions in the questionnaire were designed according to the ISPD rapid response statement [18]. It seems that this is the first time such a questionnaire was used to assess women’s knowledge on NIPT as far as we know. Whether a shorter questionnaire consisting of knowledge questions 1 to 5 is sufficient to test women’s basic knowledge on NIPT requires further study, as knowledge questions 6 to 10 appeared too complicated or difficult for the women to answer. Limitations of this pilot study are the small sample size, single centre and exclusively Chinese population. Significant bias might have been introduced by offering assistance by an experienced midwife to the women during completion of the questionnaire. However, the assistance was provided in a straightforward manner and confined to explanation of medical terms or general terms if Cantonese was not their mother tongue. Moreover, we did not assess women’s knowledge on other limitations of NIPT involving turn-around time, screening for open neural tube defects, anomaly, and late-pregnancy complications as stated in the ACMG policy statement [20]. Furthermore, we also did not assess other aspects like determination of fetal sex, maternal DNA abnormalities [38] and paternity testing [39]. In this pilot study, question 4 in Table 2 was not well constructed because it consisted of two sentences, although we intended to pose the last part; that is, for some women the extended period awaiting confirmatory invasive testing results is likely to be highly stressful. The lack of validity checking by experts, assessment of applicability and reliability of the questionnaire are limitations of this pilot study. We will further develop the questionnaire in our subsequent studies.

## Conclusions

It is feasible to use a questionnaire based on the ISPD statement on NIPT to assess women’s knowledge. The Chinese pregnant women who underwent NIPT knew more about the false positive and false negative results than the test accuracy in comparison to an invasive test, and the possibility of a non-informative report, but knew little about the more complicated aspects of NIPT. Less than half of them received information from medical professionals. More information by healthcare professionals is required for an informed choice and to avoid misunderstanding. With increasing use of NIPT in clinical practice for wider indications, it is important to find ways to keep healthcare professionals updated so that they can provide adequate information to women before testing. In Hong Kong, counselling on NIPT by trained midwives with the support of obstetricians for abnormal or difficult cases is worth further exploration. It seems that women prefer NIPT as a contingent measure to a primary screening test probably because of its high cost, but this preference may change, as the costs will likely go down in the future.

## Abbreviations

NIPT: Non-invasive prenatal testing
cffDNA: Cell-free fetal DNA
